# Symbiont modulates expression of specific gene categories in *Angomonas deanei*

**DOI:** 10.1590/0074-02760160228

**Published:** 2016-10-03

**Authors:** Luciana Loureiro Penha, Luísa Hoffmann, Silvanna Sant’Anna de Souza, Allan Cézar de Azevedo Martins, Thayane Bottaro, Francisco Prosdocimi, Débora Souza Faffe, Maria Cristina Machado Motta, Turán Péter Ürményi, Rosane Silva

**Affiliations:** Universidade Federal do Rio de Janeiro, Instituto de Biofísica Carlos Chagas Filho, Rio de Janeiro, RJ, Brasil

**Keywords:** trypanosomatid RNA-seq, gene expression, aposymbiotic strain, endosymbiosis

## Abstract

Trypanosomatids are parasites that cause disease in humans, animals, and plants. Most are non-pathogenic and some harbor a symbiotic bacterium. Endosymbiosis is part of the evolutionary process of vital cell functions such as respiration and photosynthesis. *Angomonas deanei* is an example of a symbiont-containing trypanosomatid. In this paper, we sought to investigate how symbionts influence host cells by characterising and comparing the transcriptomes of the symbiont-containing *A. deanei* (wild type) and the symbiont-free aposymbiotic strains. The comparison revealed that the presence of the symbiont modulates several differentially expressed genes. Empirical analysis of differential gene expression showed that 216 of the 7625 modulated genes were significantly changed. Finally, gene set enrichment analysis revealed that the largest categories of genes that downregulated in the absence of the symbiont were those involved in oxidation-reduction process, ATP hydrolysis coupled proton transport and glycolysis. In contrast, among the upregulated gene categories were those involved in proteolysis, microtubule-based movement, and cellular metabolic process. Our results provide valuable information for dissecting the mechanism of endosymbiosis in *A. deanei.*

An important part of eukaryotic cell evolution was the acquisition of new organelles through endosymbiosis. For example, the development of organelles with vital cell functions such as respiration and photosynthesis is a result of the mutualistic association of eukaryotic cells with bacterial ancestors that gave rise to the mitochondrion and chloroplast. The study of endosymbiosis in a primitive eukaryotic model such as a trypanosomatid may help provide insight on the fundamentals of this biological process. Trypanosomatids are known for causing diseases in humans, animals, and plants of economic importance; however, not all of these microorganisms are pathogenic. Most species of the family are monoxenic non-pathogenic-organisms that inhabit a single invertebrate host, usually an insect. An example is the wild type (WT) *Angomonas deanei* (previously classified as *Crithidia deanei*) (Teixeira et al. 2011) which hosts a betaproteobacteria of the Alcaligenacea family in its cytoplasm. This symbiosis benefits the protozoan host because the bacterium contains genes that encode enzymes that complete essential biosynthetic pathways (Alves et al. 2011, 2013, Klein et al. 2013, Motta et al. 2013). On the other hand, the aposymbiotic (APO) *A. deanei* lacks the betaproteobacteria. This cured strain was generated by chloramphenicol treatment and thus represents a valuable tool to study how the symbiont influences *A. deanei* morphology and physiology (Mundim et al. 1974, Chang 1975). An intense metabolic exchange between the bacterium and the host protozoan occurs (Motta et al. 2013). The benefits of the exchange are evidenced by the symbiont-bearing WT having reduced nutritional requirements and enhanced growth rate when compared with other protozoans of the family (Mundim et al. 1974, de Souza & Motta 1999, Frossard et al. 2006). Furthermore, we have previously shown that the presence of the symbiont modifies the surface charge, the carbohydrate composition, and ultrastructural features of the host *A. deanei* (de Souza & Motta 1999).

Genome sequence analysis and nutritional assays reveal that the symbiont complements essential biosynthetic pathways of the trypanosomatid such as the production of haeme, amino acids (aas), vitamins and purine/pyrimidine bases (Alves et al. 2011, 2013, Klein et al. 2013, Motta et al. 2013). Therefore, the endosymbiosis in this trypanosomatid constitutes a mutualistic association where participants co-evolved leading to mutual dependence in which the cured APO protozoa is unable to colonise insects and the isolated bacterium is unable to replicate in culture media (de Souza & Motta 1999).

The influence of symbionts on host gene expression has been studied in several other organisms: in flowering plants, the association with glomeromycotan fungi; in coral, the association of cnidarians with algae; and in lichens, the association with algae and fungi (Oldroyd et al. 2009, Devers et al. 2011, Junttila & Rudd 2012, Lehnert et al. 2014, Zhao et al. 2014). However, these studies are limited to taxonomically distinct groups of symbionts and carried out using either less robust large-scale RNA sequencing or only quantitative polymerase chain reaction (qPCR) techniques. Therefore, in this paper, we used a more robust large-scale RNA sequence technology to investigate how symbionts influence host cells by characterising and comparing the transcriptomes of the symbiont-containing *A. deanei* WT and the symbiont-free APO strains. The results obtained suggest that the symbiont influences *A. deanei* gene expression by upregulating or downregulating specific limited number of gene categories involved in critical cellular processes. We believe that our characterisation of the *A. deanei* transcriptome profiles provides valuable information for dissecting the mechanism of endosymbiosis in this trypanosomatid.

## MATERIALS AND METHODS

*Cell culture* - *A. deanei* WT isolated from *Zelus leucogrammus* (ATCC 30255) and APO strain (ATCC 044) were grown at 28ºC in Warren culture medium (37 g/L brain and heart infusion, 0.03 mg/L hemin, and 10 mg/L folic acid) supplemented with 10% fetal calf serum (Mundim et al. 1974) and without chloramphenicol.

*RNA isolation, analysis, library construction, and sequencing* - Total RNA was isolated from three biological replicates of mid-log phase cells (3 to 6 x 106 cells/mL) from both WT and APO strains of *A. deanei* using Trizol reagent (Invitrogen, Carlsbad, USA) according to manufacturer’s instructions. The RNA was subjected to two rounds of poly (A) selection with Micro PolyA Purist Small Scale mRNA Purification Kit (Ambion, Carlsbad, USA) according to Kolev et al. (2010). Poly (A)+ RNA quality and concentration were assessed on an Agilent 2100 Bioanalyzer (Agilent Technologies, Santa Clara, USA) using the RNA 6000 Nano Kit (Agilent). Five hundred nanograms of poly (A)+ RNA were used for RNA-seq library preparation. Sequencing was performed on an Ion Torrent Personal Genome Machine (Life Technologies, Carlsbad, USA). Briefly, poly (A)+ mRNA was fragmented using RNase III and a whole transcriptome library was constructed using the Ion Total RNA-seq Kit v2 (Life Technologies). The library was clonally amplified on Ion Sphere Particles (Life Technologies) using the Ion One Touch 200 Template Kit v2 (Life Technologies).

*Sequence data* - Sequence data from three replicate experiments were analysed using CLC Genomics Workbench v8.5 (CLC Bio, Qiagen, Aarhus, Denmark). Reads were trimmed by removal of ambiguous nucleotides, the poly (A) tail, and the mini-exon sequence. Quality was filtered for 0.05. Mapping of the reads was performed using the *A. deanei* ORFs as the reference (GenBank accession PRJNA169008) (Motta et al. 2013). The following parameters were used: mismatch cost of 2, insertion and deletion cost of 3, length fraction of 0.5, and similarity fraction of 0.8, using both strands and a maximum number of hits for a read of 10 and no global alignment.

Expression values of mRNA transcripts for each *A. deanei* strain (APO and WT) were counted as RPKM (Reads Per gene Kb per million of Mapped reads (Mortazavi et al. 2008). Data are available as SRA Accession: PRJNA279893.

*Differential gene expression analysis* - The CLC Genomics Workbench’s Empirical analysis of differential gene expression (EDGE) (Robinson et al. 2010) was used to compare the transcriptome profiles of APO and WT strains. The following parameters were used: common dispersion of 1e-14, total count cut-off of 5, estimate tag-wise dispersion and false discovery rate (FDR) ≤ 0.05.

*Gene set enrichment analysis* - Enrichment of functional gene categories in APO strain in relation to WT was obtained by gene set enrichment analysis (GSEA) (Tian et al. 2005). GSEA is able to associate differentially expressed gene sets with specific functional gene categories based on gene ontology (GO) terms. For each annotation category, GSEA investigates whether the ranks of the gene sets in the group are evenly spread throughout the ranked list or tend to occur at the top or bottom of the list far from a normal distribution. For each category, the lower and upper tail probabilities are calculated by comparing the original category test statistics to the distribution of the permutation-based test statistics for that category. Unique annotated sequences were analysed, and 10,000 permutations were used for permutation-based p-value calculations. Differentially expressed gene features were determined by standard t-test statistic. Gene-sets with a false discovery rate ≤ 0.05 considered significant.

## RESULTS AND DISCUSSION

*Number and abundance of transcripts in A. deanei in the absence and in the presence of the symbiont* - RNA sequencing libraries (RNA-seq) were constructed from poly (A)+ RNA extracted from three independent biological replicates of APO and WT strains of *A. deanei* on exponential growth phase.

The RNA-seq results revealed a total of 10,263,622 trimmed reads (1.3 Gb): 5,681,608 reads from APO cells and 4,582,014 reads from WT cells. The scale sequencing obtained, ~10 million reads, was sufficient to construct transcriptome profiles of the strains. The reads were mapped to the sequence of the *A. deanei* nuclear genome obtained by Motta et al. (2013). The abundance of each transcript may be calculated based on the normalisation of its gene length in relation to the total number of its reads mapped on the genome by reads per kilobase per million mapped reads (RPKM). The individual transcriptome analyses by RPKM among the 100 most abundant RNAs found in the absence (APO) and in the presence of the symbiont (WT strain) revealed a similar distribution of transcript abundance between the two strains. Among the 100 most abundant transcripts in both APO and WT strains (Supplementary data, Table I) were: (i) housekeeping transcritpts for translational and ribosomal proteins; (ii) surface amastin transcript for a transmembrane glycoprotein of trypanosomatids (Jackson 2010); (iii) membrane protein transcript for protein 11 (KMP-11), a major cell surface glycoprotein associated with membrane structures (Stebeck et al. 1995); (iv) structural protein transcripts for tubulins (Rondinelli et al. 1986); (v) DNA-associated protein transcripts for histones; and (vi) cellular metabolism protein transcripts for beta-fructofuranosidase (sugar metabolism), glyceraldehyde-3-phosphate dehydrogenase (a well-conserved glycosomal enzyme) (Hannaert et al. 1998), and transcript for s-adenosylmethionine synthetase (catalyses the formation of S-adenosylmethionine). In essence, the RPKM transcriptome analyses revealed that either in the absence of the symbiont (APO) or in the presence of the symbiont (WT) the organisms had a similar general expression pattern upon the comparison of the 100 most abundant transcripts (Supplementary data, Table I). Basically, these transcripts were the same most abundant ones found in the transcriptome profile of *Leishmania* (Rastrojo et al. 2013).

The existence of a group of transcripts that constitutes a common profile, i.e., a transcriptome signature of the Trypanosomatid family, supports previous findings of a core genome of trypanosomatids (El-Sayed et al. 2005).

**Fig. 1:**
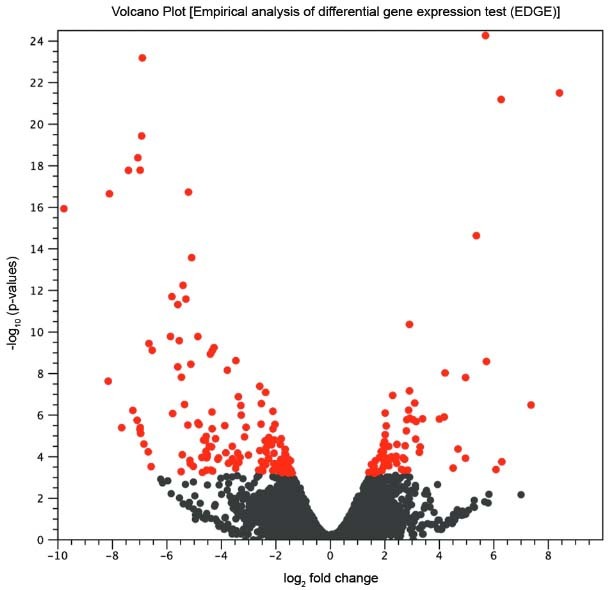
: volcano plot of empirical analysis of differential gene expression (EDGE) of *Angomonas deanei* aposymbiotic (APO) and wild type (WT) strains (three replicates each). The X-axis is the fold change (log2 scale) of up and downregulated genes in APO compared to WT strain. The Y-axis represents the p-value (log10) for the statistical test of differences between the samples (APO and WT). The red dots represent the genes that were downregulated (145) and upregulated (71) in APO strain in relation to WT strain, with false discovery rate (FDR) ≤ 0.05 and fold change > 1.5 fold of absolute values.

*Differentially expressed transcripts in A. deanei in the absence and in the presence of the symbiont identified by comparison of transcriptome profiles* - To identify differentially expressed genes in *A. deanei* triggered by the presence of the symbiotic bacterium the transcriptome profiles of *A. deanei* APO and WT strains were compared by EDGE. EDGE is a methodology based on a two-group comparison developed to study large data, such as gene expression profiles, from experiments such as RNA-seq. EDGE allows the measurement of fold change levels of transcript abundances differentially expressed on replicate data sets between two RNA populations. The EDGE comparison between the transcriptome profiles of APO and WT strains in relation to negative fold change abundance (i.e., downregulation) and positive fold change abundance (i.e., upregulation), both of at least 1.5 times, are shown in the volcano plot in Fig. 1. A total of 7625 gene transcripts in APO strain (red and gray dots in Fig. 1) were either downregulated (2108 transcripts) or upregulated (5517 transcripts). However, only a small fraction of the 7625 modulated gene transcripts, i.e., 216 genes, showed significant p-values < 0.05 (red dots in Fig. 1) of fold changes. There were 145 downregulated genes (negative values of log2 fold change, red dots), and 71 upregulated genes (positive values of log2 fold change, red dots). The identities and the fold change in abundance of specific transcripts significantly downregulated or upregulated between the two strains (APO and WT) (Supplementary data, Tables II-III, respectively). The X-axis, on a log2 scale, is the fold change of up and downregulated genes in APO compared to WT strain. The Y-axis, on log10, represents the p-value for the statistical test of differences between the samples (APO and WT). The red dots represent genes with false discovery rate (FDR) ≤ 0.05.

The downregulated genes in the APO strain (Supplementary data, Table II) were those involved in glycosomal and mitochondrial metabolism as well as those involved in the biosynthesis of nutritional elements such as aas, lipids, carbohydrates, and nucleotides. Concerning glycosomal metabolism in the APO strain, downregulation of transcripts of glycolytic enzymes was observed. One example is phosphoenolpyruvate carboxykinase (gPEPCK), which is involved in carbon removal in the citric acid cycle for various biosynthetic and oxidative processes. The findings are consistent with the high biosynthesis of aas and nucleotides observed in symbiont-harboring trypanosomatids (Mundim et al. 1974, Klein et al. 2013, Motta et al. 2013). Comparatively, in another symbiosis, such as in ectomycorrhizal, the transcriptome analysis shows that the presence of a soil fungus alters the transcription of genes also involved in plant glycolysis with upregulation of malate dehydrogenase and downregulation of pyruvate kinase (Sebastiana et al. 2014). In the present study, other downregulated enzymes in APO strain were detected such as aconitase and mitochondrial isocitrate dehydrogenase. These enzymes are involved in citrate metabolism which plays a major role in symbiosis relationship through iron transportation as is found in legume-rhizobium symbiosis (Brear et al. 2013). On the other hand, the upregulated genes in the APO cells (Supplementary data, Table III) included genes for proteases (as serine peptidase and metacaspase), heat shock protein 100, mitochondrial tryparedoxin, vacuolar protein sorting-associated protein 4, protein kinase, branched-chain amino acid (aa) aminotransferase, minichromosome maintenance complex, and formin. The observed upregulation of mitochondrial tryparedoxin transcript (TXN) in the APO cells may play a role in parasite protection against oxidative stress. TXN usually is required for trypanosomatid metabolism. Nevertheless, knout-out of the gene in *Leishmania infantum* (LiTXN2) does not promote a significant effect on parasite survival (Castro et al. 2010). In conclusion, the observation of up or down regulation of specific gene categories in the APO strain suggests that the endosymbiont interferes in the gene expression of several host cell processes.

**Fig. 2:**
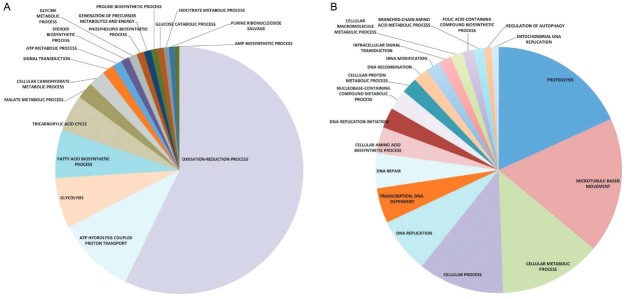
: enrichment of genes from functional categories that are significantly downregulated (A) and upregulated (B) in aposymbiotic *Angomonas deanei* (APO) strain using the wild type strain as a reference. biological process using gene ontology (GO) categorised genes into broader groups. Representative GO terms of biological function and number of genes involved in each function are indicated in Supplementary data, Tables IV-V.

*Identification of specific groups of genes enriched in A. deanei in the absence and in the presence of the symbiont* - To better identify groups of genes associated with the presence of the endosymbiont and that may be responsible for given functional pathways enrichment analysis of functional category genes (GSEA) was performed in APO and the WT strain. GSEA measures differential expression of genes as EDGE does. However, GSEA is able to associate differentially expressed gene sets with specific functional gene categories based on GO terms. GO is a well-ordered lexicon of terms that defines groups of gene products related to biological processes, molecular functions, and cellular components. Supplementary data, Tables IV-V, show the results of GSEA for the differentially expressed functional category of enrichment of genes involved in biological processes that were down and upregulated, respectively, in the APO strain. Gene categories were ordered according to their statistical p-value (≤ 0.05) in the GSEA. A total of 18 categories of genes involved in biological processes were downregulated in the APO strain (Supplementary data, Table IV). If the categories with a higher number of genes are considered, an overall impression can be obtained of the most represented set of differentially expressed genes downregulated in the APO strain. The most represented gene sets down-regulated in biological processes listed in Supplementary data, Table IV, were: oxidation-reduction (418 genes), ATP hydrolysis coupled proton transport (75 genes), glycolysis (48 genes), fatty acid biosynthesis (46 genes), tricarboxylic acid cycle (35 genes), malate metabolism (16 genes), carbohydrate metabolism (16 genes), and signal transduction (11 genes). Conversely, 19 categories of genes involved in biological processes were upregulated in the APO strain (Supplementary data, Table V). Using the same parameters as above, the most represented gene sets upregulated in biological processes listed in Supplementary data, Table V, were: proteolysis (95 genes), microtubule-based movement (93 genes), cellular metabolic process (70 genes), cellular process (59); DNA replication (38 genes), DNA-dependent transcription (24 genes), cellular aa (18 genes), DNA replication initiation (14 genes), nucleobase-containing compound metabolic process (14 genes), and cellular protein metabolic process (11 genes). In addition to the biological processes category described above, other gene categories such as those involved in cellular components, and those involved in molecular functions were influenced similarly by the presence of the endosymbiont (data not shown). To simplify the data presentation in Supplementary data, Tables IV-V, the data also are presented in a pie chart format (Fig. 2A-B, respectively). It became evident that the largest category of genes down-regulated in the absence of the symbiont (APO strain) was by far the one involved in oxidation-reduction process. ATP hydrolysis coupled proton transport and glycolysis were the next largest categories (Fig. 2A). In contrast, among the up-regulated category of genes, also in the absence of the symbiont (Fig. 2B), three categories stood out: proteolysis, microtubule-based movement, and cellular metabolic process. Several other groups, contributing a smaller number of genes, also were down or upregulated (Fig. 2A-B). The influence of symbionts in host gene expression has been shown in other symbiotic associations (Lehnert et al. 2012, 2014, Kodama et al. 2014, Zhao et al. 2014). Comparative transcriptional analysis performed in orchid *Cymbidium hybridum* in the presence or absence of the mycorrhizal fungus showed that the symbiont positively influences plant gene expression. Genes involved in reactive oxygen species detoxification such as phosphate transport, cell wall synthesis, root morphogenesis, and cell signaling are all influenced by the symbiont (Zhao et al. 2014). The influence of the bacterium symbiont in the host *A. deanei*, as pointed out in this work, also occurs by modulation of expression of specific gene categories. This influence is achieved by up or downregulating the expression of specific genes. If the most represented categories arbitrarily considered as the most relevant, then, among the downregulated will be those for oxidation-reduction process, ATP hydrolysis coupled proton transport, and glycolysis. On the other hand, among the upregulated genes will be those for proteolysis, microtubule-based movement, and cellular metabolic process. However, the possibility that less represented categories may be equally important cannot be ignored. Nevertheless, the data offer now clear evidence for selecting specific types of genes, and their protein products, to be further studied to dissect the complex interaction of the two organisms. Hopefully, the data also may contribute to unraveling the origin of various organelles in eukaryotic cells. At this point, one may speculate based on the results herein as well as other groups’ results. For example, considering genes for the energetic metabolism that requires functioning of the mitochondrion and the glycosome, it already has been established that the symbiont-containing *A. deanei* presents higher phosphorylation capacity and oxygen consumption than does APO cells (Azevedo-Martins et al. 2015). In agreement with this finding, the genes for oxidative phosphorylation, ATP hydrolysis coupled to proton transport and Krebs cycle were downregulated in APO cells. This observation may constitute direct evidence of the well-succeeded symbiosis between the bacterium and the insect parasite *A. deanei*. Although the results herein covered the majority of expressed genes, further work should be carried out using more extensive high RNA-seq data and a larger number of replicates to identify genes expressed at very low levels. Finally, proteome profile analysis of the products encoded by the gene categories pointed out in this work should be carried out to investigate their roles in the mechanisms involved in this symbiotic process.

In summary, the question of how symbionts influence the host cells was assessed through characterisation and comparison of the transcriptomes of symbiont-containing *A. deanei* (WT) and symbiont-free APO strains using robust large-scale RNA sequencing technology. In essence, the organisms have a similar general expression pattern based on a comparison of the 100 most abundant transcripts. Interestingly, this set of transcripts is the same set of most abundant transcripts found in the transcriptome of *Leishmania*. This finding supports the existence of a group of transcripts that constitutes a common profile, i.e., a transcriptome signature of the trypanosomatid family. Finally, the results show that the symbiont influences the host *A. deanei* by modulating gene expression of specific genes. The influence is achieved by up or downregulating the expression of selected categories of genes involved in crucial cellular mechanisms such as oxidation-reduction process, ATP hydrolysis coupled proton transport, glycolysis, proteolysis, microtubule-based movement, and cellular metabolic process. Future studies focusing on these gene categories may increase our understanding of the symbiotic process in *A. deanei.*
