# Supplementary material for: Symbiont modulates expression of specific gene categories in Angomonas deanei
Source: Mem Inst Oswaldo Cruz. 2016 Oct 3;111(11):686–91. doi: 10.1590/0074-02760160228 (PMC5125052; doi:10.1590/0074-02760160228)
Supplement: Supplementary file 1 [file 0074-0276-mioc-0074-02760160228-suppl01.pdf]

*Mem Inst Oswaldo Cruz*, Rio de Janeiro, Vol. *111*(11): 1-7, November 2016 

TABLE I

The 100 most abundant transcripts of both wild type (WT) and aposymbiotic (APO) strains of *Angomonas deanei*, measured as RPKM

| | Feature ID | Gene function (Annotation) | WT transcript abundance (RPKM - Mean) | Feature ID | Gene function (Annotation) | APO transcript abundance (RPKM - Mean) |
|---|---|---|---|---|---|---|
| 1 | AGDE17275 | hypothetical protein | 39,310 | AGDE17274 | hypothetical protein | 25,210 |
| 2 | AGDE17274 | hypothetical protein | 25,847 | AGDE17275 | hypothetical protein | 24,695 |
| 3 | AGDE10555 | beta-fructofuranosidase | 7,347 | AGDE14376 | hypothetical protein | 8,569 |
| 4 | AGDE12097 | glycosomal glyceraldehyde-3-phosphate partial | 5,267 | AGDE10555 | beta-fructofuranosidase | 7,893 |
| 5 | AGDE00565 | kinetoplastid membrane protein 11 | 5,265 | AGDE13194 | hypothetical protein | 7,657 |
| 6 | AGDE01837 | 60s ribosomal protein l22 | 4,916 | AGDE00565 | kinetoplastid membrane protein 11 | 6,129 |
| 7 | AGDE05651 | 40s ribosomal protein s3a | 4,858 | AGDE14377 | hypothetical protein | 5,952 |
| 8 | AGDE12439 | 40s ribosomal protein s23 | 4,795 | AGDE03921 | s-adenosylmethionine synthetase | 5,733 |
| 9 | AGDE08950 | hypothetical protein | 4,077 | AGDE15019 | hypothetical protein | 5,230 |
| 10 | AGDE02419 | glycosomal glyceraldehyde-3-phosphate partial | 4,058 | AGDE08950 | hypothetical protein | 5,130 |
| 11 | AGDE09629 | pyruvate indole-pyruvate carboxylase | 3,955 | AGDE05874 | calcium-binding protein | 4,972 |
| 12 | AGDE13194 | hypothetical protein | 3,922 | AGDE01203 | glycosomal phosphoenolpyruvate carboxykinase | 4,122 |
| 13 | AGDE11644 | translation elongation factor 1-beta | 3,900 | AGDE09744 | calcium-binding protein | 4,062 |
| 14 | AGDE03921 | s-adenosylmethionine synthetase | 3,865 | AGDE11644 | translation elongation factor 1-beta | 3,835 |
| 15 | AGDE05874 | calcium-binding protein | 3,741 | AGDE07510 | calcium-binding protein | 3,808 |
| 16 | AGDE08485 | 60s acidic ribosomal subunit protein | 3,594 | AGDE08517 | s-adenosylmethionine synthetase | 3,705 |
| 17 | AGDE15019 | hypothetical protein | 3,567 | AGDE07844 | calcium-binding protein | 3,696 |
| 18 | AGDE10788 | eukaryotic initiation factor 5a | 3,566 | AGDE05651 | 40s ribosomal protein s3a | 3,598 |
| 19 | AGDE14377 | hypothetical protein | 3,477 | AGDE01837 | 60s ribosomal protein l22 | 3,525 |
| 20 | AGDE00325 | histone h4 | 3,410 | AGDE08485 | 60s acidic ribosomal subunit protein | 3,294 |
| 21 | AGDE09744 | calcium-binding protein | 3,126 | AGDE12439 | 40s ribosomal protein s23 | 3,142 |
| 22 | AGDE02676 | 40s ribosomal protein s9 | 3,079 | AGDE02676 | 40s ribosomal protein s9 | 3,096 |
| 23 | AGDE09466 | beta-fructofuranosidase | 3,064 | AGDE12685 | 40s ribosomal protein | 3,048 |
| 24 | AGDE05681 | 40s ribosomal protein | 3,005 | AGDE14768 | conserved hypothetical protein | 2,989 |
| 25 | AGDE07844 | calcium-binding protein | 2,853 | AGDE09629 | pyruvate indole-pyruvate carboxylase | 2,986 |
| 26 | AGDE10521 | cysteine peptidase | 2,851 | AGDE13022 | amastin | 2,879 |
| 27 | AGDE15370 | myo-inositol-1-phosphate synthase | 2,830 | AGDE03102 | 40s ribosomal protein l14 | 2,853 |
| 28 | AGDE08517 | s-adenosylmethionine synthetase | 2,788 | AGDE10788 | eukaryotic initiation factor 5a | 2,697 |
| 29 | AGDE04958 | ubiquitin ribosomal protein s27a | 2,730 | AGDE03560 | calpain-like cysteine peptidase | 2,673 |
| 30 | AGDE01084 | eukaryotic initiation factor 5a | 2,687 | AGDE03923 | 60s ribosomal protein l11 ( l16) | 2,670 |
| 31 | AGDE14376 | hypothetical protein | 2,679 | AGDE05681 | 40s ribosomal protein | 2,668 |
| 32 | AGDE02314 | 40s ribosomal protein s13 | 2,677 | AGDE02314 | 40s ribosomal protein s13 | 2,657 |
| 33 | AGDE13022 | amastin | 2,644 | AGDE05973 | 60s ribosomal protein l19 | 2,633 |
| 34 | AGDE10805 | 40s ribosomal protein | 2,605 | AGDE00500 | calpain-like cysteine peptidase | 2,606 |

| | Feature ID | Gene function (Annotation) | WT transcript abundance (RPKM - Mean) | Feature ID | Gene function (Annotation) | APO transcript abundance (RPKM - Mean) |
|---|---|---|---|---|---|---|
| 35 | AGDE07943 | hypothetical protein | 2,536 | AGDE11775 | 60s ribosomal protein l7a | 2,551 |
| 36 | AGDE14768 | conserved hypothetical protein | 2,528 | AGDE09466 | beta-fructofuranosidase | 2,438 |
| 37 | AGDE12685 | 40s ribosomal protein | 2,504 | AGDE00524 | aldehyde mitochondrial precursor | 2,375 |
| 38 | AGDE08044 | protein disulfide isomerase | 2,470 | AGDE04284 | glutamate dehydrogenase | 2,302 |
| 39 | AGDE03793 | ribosomal protein s29 | 2,421 | AGDE10494 | ama1 | 2,292 |
| 40 | AGDE07510 | calcium-binding protein | 2,401 | AGDE07943 | hypothetical protein | 2,266 |
| 41 | AGDE15443 | g-amastin | 2,389 | AGDE10521 | myristoylated protein 3 | 2,264 |
| 42 | AGDE07941 | elongation factor 1-alpha | 2,377 | AGDE04196 | 60s acidic ribosomal protein p2 | 2,230 |
| 43 | AGDE02059 | 60s ribosomal protein l27a l29 | 2,364 | AGDE03670 | 60s ribosomal protein l18a | 2,230 |
| 44 | AGDE05615 | 40s ribosomal protein s15a | 2,326 | AGDE12965 | d-amastin | 2,202 |
| 45 | AGDE03670 | 60s ribosomal protein l18a | 2,294 | AGDE01084 | eukaryotic initiation factor 5a | 2,194 |
| 46 | AGDE00500 | calpain-like cysteine peptidase | 2,289 | AGDE10805 | 40s ribosomal protein | 2,187 |
| 47 | AGDE14033 | hypothetical protein | 2,283 | AGDE04017 | proton motive atpase 1 | 2,166 |
| 48 | AGDE00524 | aldehyde mitochondrial precursor | 2,251 | AGDE02155 | heat shock protein 70 | 2,134 |
| 49 | AGDE04017 | proton motive atpase 1 | 2,245 | AGDE15370 | myo-inositol-1-phosphate synthase | 2,124 |
| 50 | AGDE04779 | argininosuccinate synthase | 2,245 | AGDE12097 | glycosomal glyceraldehyde-3-phosphate partial | 2,111 |
| 51 | AGDE04193 | alcohol dehydrogenase | 2,210 | AGDE04958 | ubiquitin ribosomal protein s27a | 2,082 |
| 52 | AGDE06563 | 60s ribosomal protein l7a | 2,206 | AGDE07674 | p-type h+-atpase | 2,059 |
| 53 | AGDE09068 | inositol-3-phosphate synthase | 2,197 | AGDE03780 | 40s ribosomal protein s15a | 2,055 |
| 54 | AGDE03560 | calpain-like cysteine peptidase | 2,133 | AGDE07941 | elongation factor 1-alpha | 2,027 |
| 55 | AGDE01203 | glycosomal phosphoenolpyruvate carboxykinase | 2,108 | AGDE09563 | polyubiquitin | 1,993 |
| 56 | AGDE16970 | hypothetical protein | 2,046 | AGDE00325 | histone h4 | 1,988 |
| 57 | AGDE03102 | 40s ribosomal protein l14 | 2,028 | AGDE07279 | heat-shock protein hsp70 | 1,970 |
| 58 | AGDE07934 | atp-dependent phosphofructokinase | 2,023 | AGDE05482 | ribosomal protein s20 | 1,965 |
| 59 | AGDE04235 | beta tubulin | 2,000 | AGDE05221 | ribosomal protein s20 | 1,955 |
| 60 | AGDE04196 | 60s acidic ribosomal protein p2 | 1,987 | AGDE06563 | 60s ribosomal protein l7a | 1,940 |
| 61 | AGDE06523 | hypothetical protein | 1,957 | AGDE14375 | circumsporozoite protein | 1,910 |
| 62 | AGDE05482 | ribosomal protein s20 | 1,936 | AGDE03362 | alpha tubulin | 1,904 |
| 63 | AGDE05221 | ribosomal protein s20 | 1,932 | AGDE10549 | glycosomal phosphoenolpyruvate carboxykinase | 1,880 |
| 64 | AGDE05973 | 60s ribosomal protein l19 | 1,932 | AGDE01346 | hypothetical protein | 1,839 |
| 65 | AGDE12965 | d-amastin | 1,907 | AGDE14033 | hypothetical protein | 1,808 |
| 66 | AGDE02155 | heat shock protein 70 | 1,903 | AGDE09786 | activated protein kinase c receptor | 1,794 |
| 67 | AGDE07477 | pyruvate indole-pyruvate carboxylase | 1,901 | AGDE03793 | ribosomal protein s29 | 1,768 |
| 68 | AGDE08102 | 40s ribosomal protein s12 | 1,884 | AGDE17226 | nucleoside diphosphate kinase b | 1,755 |
| 69 | AGDE00554 | 40s ribosomal protein s12 | 1,856 | AGDE13180 | hypothetical protein | 1,725 |
| 70 | AGDE10067 | fructose-bisphosphate glycosomal | 1,853 | AGDE07979 | 60s ribosomal protein l11 ( l16) | 1,722 |

| | Feature ID | Gene function (Annotation) | WT transcript abundance (RPKM - Mean) | Feature ID | Gene function (Annotation) | APO transcript abundance (RPKM - Mean) |
|---|---|---|---|---|---|---|
| 71 | AGDE03780 | 40s ribosomal protein s15a | 1,848 | AGDE06789 | 60s ribosomal protein l27a l29 | 1,696 |
| 72 | AGDE07738 | 60s ribosomal protein l18a | 1,847 | AGDE08044 | protein disulfide isomerase | 1,653 |
| 73 | AGDE10417 | activated protein kinase c receptor | 1,832 | AGDE04779 | argininosuccinate synthase | 1,653 |
| 74 | AGDE03362 | alpha tubulin | 1,820 | AGDE13179 | hypothetical protein | 1,625 |
| 75 | AGDE07283 | calpain-like cysteine peptidase | 1,809 | AGDE15968 | 40s ribosomal protein s21 | 1,602 |
| 76 | AGDE09786 | activated protein kinase c receptor | 1,799 | AGDE04235 | beta tubulin | 1,584 |
| 77 | AGDE05045 | 60s ribosomal protein l36 | 1,735 | AGDE07283 | calpain-like cysteine peptidase | 1,579 |
| 78 | AGDE04641 | cyclophilin a | 1,729 | AGDE05615 | 40s ribosomal protein s15a | 1,574 |
| 79 | AGDE17226 | nucleoside diphosphate kinase b | 1,711 | AGDE02419 | glycosomal glyceraldehyde-3-phosphate partial | 1,562 |
| 80 | AGDE02533 | 40s ribosomal protein s11 | 1,707 | AGDE05634 | ubiquitin ribosomal protein s27a | 1,550 |
| 81 | AGDE00667 | 60s ribosomal protein l36 | 1,686 | AGDE02152 | 40s ribosomal protein | 1,549 |
| 82 | AGDE04164 | iron superoxide dismutase | 1,654 | AGDE03620 | argininosuccinate synthase | 1,549 |
| 83 | AGDE07279 | heat-shock protein hsp70 | 1,648 | AGDE13309 | hypothetical protein | 1,519 |
| 84 | AGDE06596 | ribosomal protein l1a | 1,626 | AGDE13304 | high cysteine membrane protein group 2 | 1,515 |
| 85 | AGDE14034 | hypothetical protein | 1,608 | AGDE09570 | hypothetical protein | 1,503 |
| 86 | AGDE15968 | 40s ribosomal protein s21 | 1,606 | AGDE11697 | 60s ribosomal protein | 1,458 |
| 87 | AGDE11342 | fructose-bisphosphate glycosomal | 1,600 | AGDE08102 | 40s ribosomal protein s12 | 1,452 |
| 88 | AGDE02152 | 40s ribosomal protein | 1,595 | AGDE11094 | 40s ribosomal protein s10 | 1,435 |
| 89 | AGDE06376 | 40s ribosomal protein s24e | 1,594 | AGDE09758 | pyruvate phosphate dikinase | 1,435 |
| 90 | AGDE10494 | ama1 protein | 1,572 | AGDE06523 | hypothetical protein | 1,432 |
| 91 | AGDE10551 | ribosomal protein s25 | 1,568 | AGDE12914 | hypothetical protein | 1,406 |
| 92 | AGDE09563 | polyubiquitin | 1,565 | AGDE07477 | pyruvate indole-pyruvate carboxylase | 1,383 |
| 93 | AGDE06789 | 60s ribosomal protein l27a l29 | 1,549 | AGDE00554 | 40s ribosomal protein s12 | 1,373 |
| 94 | AGDE11094 | 40s ribosomal protein s10 | 1,543 | AGDE02059 | 60s ribosomal protein l27a l29 | 1,347 |
| 95 | AGDE07979 | 60s ribosomal protein l11 (l16) | 1,522 | AGDE10678 | hypothetical protein | 1,346 |
| 96 | AGDE07674 | p-type h+-atpase | 1,497 | AGDE15802 | hypothetical protein | 1,333 |
| 97 | AGDE08528 | 60s ribosomal subunit protein l31 | 1,493 | AGDE05636 | 60s ribosomal protein l28 | 1,320 |
| 98 | AGDE04284 | glutamate dehydrogenase | 1,492 | AGDE04499 | ubiquitin-fusion protein | 1,315 |
| 99 | AGDE03173 | 60s ribosomal protein l22 | 1,479 | AGDE13186 | hypothetical protein | 1,301 |
| 100 | AGDE03923 | 60s ribosomal protein l11 ( l16) | 1,450 | AGDE01887 | ribosomal protein l15 | 1,301 |

Expression means of the 100 most abundant transcripts quantified by reads per gene kb per million of mapped reads (RPKM) obtained from three independent biological replicates from aposymbiotic (APO) strain and wild type (WT) strain of *Angomonas deanei* on exponential growth. AGDE: *A. deanei* sequence feature (SRA Accession: PRJNA279893).

TABLE II

Differential gene expression between *Angomonas deanei* aposymbiotic (APO) and wild type (WT) strains. List of gene transcripts down regulated in APO strain

| Feature ID | Fold change | Gene function (Annotation) | FDR p-value |
|---|---|---|---|
| AGDE02219 | -119 | ribosomal protein l27 | 5.5E-20 |
| AGDE01379 | -122 | aconitase | 1.2E-16 |
| AGDE04780 | -134 | glycosomal phosphoenolpyruvate carboxykinase | 1.2E-15 |
| AGDE11451 | -170 | delta-1-pyrroline-5-carboxylate dehydrogenase | 3.5E-15 |
| AGDE00951 | -126 | myosin XXI | 3.5E-15 |
| AGDE01738 | -37 | pyrroline-5-carboxylate synthetase-like protein | 3.5E-14 |
| AGDE05250 | -276 | serine threonine kinase-like protein | 3.8E-14 |
| AGDE04771 | -877 | elongation factor tu | 1.8E-13 |
| AGDE07812 | -34 | 60s ribosomal protein | 3.4E-11 |
| AGDE12291 | -42 | conserved hypothetical protein | 6.9E-10 |
| AGDE13031 | -56 | poly ADP-ribose polymerase | 2.2E-09 |
| AGDE16801 | -39 | hypothetical protein | 2.7E-09 |
| AGDE01355 | -48 | sre-2/carboxylase carrier protein | 4.7E-09 |
| AGDE00619 | -58 | profilin | 1.4E-07 |
| AGDE09078 | -29 | squalene synthase | 1.4E-07 |
| AGDE12173 | -47 | conserved hypothetical protein | 2.1E-07 |
| AGDE09437 | -101 | serine threonine protein phosphatase catalytic subunit | 2.7E-07 |
| AGDE10173 | -19 | conserved hypothetical protein | 4.1E-07 |
| AGDE11664 | -93 | glycosomal phosphoenolpyruvate carboxykinase | 5.3E-07 |
| AGDE06268 | -20 | dihydrolipoamide dehydrogenase | 5.5E-07 |
| AGDE07437 | -21 | p450 reductase | 7.5E-07 |
| AGDE17104 | -11 | rhodanase-like protein | 1.5E-06 |
| AGDE16921 | -35 | conserved hypothetical protein | 2.1E-06 |
| AGDE06828 | -48 | vacuolar atp synthase subunit c | 2.7E-06 |
| AGDE08262 | -14 | conserved hypothetical protein | 3.8E-06 |
| AGDE12242 | -44 | conserved hypothetical protein | 7.6E-06 |
| AGDE16972 | -284 | hypothetical protein | 1.1E-05 |
| AGDE07356 | -6 | camp specific phosphodiesterase | 1.9E-05 |
| AGDE06718 | -5 | P27 protein | 3.6E-05 |
| AGDE10206 | -10 | 2-oxoglutarate e3 -like protein | 5.5E-05 |
| AGDE03552 | -6 | coproporphyrinogen iii oxidase | 0.0001 |
| AGDE07260 | -41 | d-3-phosphoglycerate dehydrogenase-like protein | 0.0001 |
| AGDE17201 | -10 | high cysteine membrane protein | 0.0001 |
| AGDE03214 | -152 | conserved hypothetical protein | 0.0002 |
| AGDE05533 | -4 | camp specific phosphodiesterase | 0.0002 |
| AGDE03177 | -20 | proteasome beta 3 subunit | 0.0002 |
| AGDE00606 | -55 | conserved hypothetical protein | 0.0003 |
| AGDE08631 | -10 | 40s ribosomal protein s8 | 0.0003 |
| AGDE17124 | -136 | hypothetical protein | 0.0005 |
| AGDE07145 | -29 | conserved hypothetical protein | 0.0007 |
| AGDE07360 | -29 | u2 splicing auxiliary factor | 0.0007 |
| AGDE12090 | -6 | aldose 1 epimerase | 0.0007 |
| AGDE04864 | -4 | sterol 24-c-methyltransferase | 0.0007 |
| AGDE07550 | -28 | conserved hypothetical protein | 0.0007 |
| AGDE09287 | -38 | udp-glucose pyrophosphorylase | 0.0008 |
| AGDE06160 | -15 | n-myristoyl transferase | 0.0008 |
| AGDE17058 | -9 | hypothetical protein | 0.0009 |
| AGDE17084 | -202 | hypothetical protein | 0.0010 |
| AGDE16802 | -126 | hypothetical protein | 0.0010 |
| AGDE05062 | -20 | conserved hypothetical protein | 0.0011 |
| AGDE03215 | -4 | coproporphyrinogen iii oxidase | 0.0011 |
| AGDE01118 | -127 | conserved hypothetical protein | 0.0011 |
| AGDE14744 | -125 | FAD linked oxidase | 0.0017 |
| AGDE06230 | -24 | glycosomal phosphoenolpyruvate carboxykinase | 0.0022 |
| AGDE10976 | -9 | metal-ion transporter | 0.0023 |
| AGDE07908 | -5 | mitochondrial isocitrate dehydrogenase | 0.0025 |
| AGDE11864 | -19 | cyclin 9 | 0.0028 |
| AGDE08758 | -3 | conserved hypothetical protein | 0.0028 |
| AGDE03939 | -4 | mitochondrial isocitrate dehydrogenase | 0.0031 |
| AGDE06048 | -25 | conserved hypothetical protein | 0.0031 |
| AGDE07651 | -24 | rna-binding protein | 0.0032 |
| AGDE00952 | -5 | aldose 1 epimerase | 0.0033 |
| AGDE11769 | -5 | squalene monooxygenase-like protein | 0.0033 |
| AGDE17193 | -115 | conserved hypothetical protein | 0.0046 |
| AGDE06650 | -4 | mitochondrial phosphate transporter | 0.0047 |
| AGDE02757 | -4 | a chain radiation-reduced tryparedoxin-i | 0.0048 |
| AGDE03018 | -5 | rad51 protein | 0.0051 |
| AGDE02649 | -22 | ethanolamine phosphotransferase | 0.0054 |
| AGDE17249 | -12 | prostaglandin f synthase | 0.0054 |
| AGDE10667 | -21 | conserved hypothetical protein | 0.0056 |
| AGDE10579 | -5 | ubiquitin hydrolase | 0.0065 |

| Feature ID | Fold change | Gene function (Annotation) | FDR p-value |
|---|---|---|---|
| AGDE09555 | -3 | cytochrome p450-like protein | 0.0071 |
| AGDE03011 | -6 | coproporphyrinogen iii oxidase | 0.0080 |
| AGDE02136 | -24 | glycosomal phosphoenolpyruvate carboxykinase | 0.0086 |
| AGDE16194 | -103 | transcriptional regulator | 0.0089 |
| AGDE05429 | -5 | conserved hypothetical protein | 0.0091 |
| AGDE00762 | -14 | conserved hypothetical protein | 0.0097 |
| AGDE00627 | -11 | lysophospholipase | 0.0099 |
| AGDE00497 | -4 | mitogen-activated protein kinase 3 | 0.0100 |
| AGDE05100 | -3 | mitogen-activated protein kinase | 0.0100 |
| AGDE07028 | -23 | conserved hypothetical protein | 0.0112 |
| AGDE16193 | -43 | hypothetical protein | 0.0116 |
| AGDE07816 | -8 | protein kinase | 0.0118 |
| AGDE06949 | -3 | fatty acid hydrolase | 0.0124 |
| AGDE03376 | -23 | 40s ribosomal protein s15 | 0.0127 |
| AGDE01304 | -25 | conserved hypothetical protein | 0.0140 |
| AGDE06436 | -26 | conserved hypothetical protein | 0.0142 |
| AGDE07443 | -10 | hypothetical protein, unknown function | 0.0142 |
| AGDE14046 | -3 | folic acid/methotrexate transporter | 0.0142 |
| AGDE09900 | -23 | chaperonin mitochondrial precursor | 0.0145 |
| AGDE00411 | -17 | proteasome regulatory non-atp-ase subunit | 0.0145 |
| AGDE08887 | -5 | conserved hypothetical protein | 0.0149 |
| AGDE02570 | -12 | protein kinase | 0.0155 |
| AGDE04064 | -5 | cytochrome b-domain protein | 0.0157 |
| AGDE06428 | -3 | glutamamyl carboxypeptidase | 0.0157 |
| AGDE06369 | -18 | conserved hypothetical protein | 0.0159 |
| AGDE07859 | -4 | ribosomal protein l1a | 0.0159 |
| AGDE17113 | -3 | rhodanese-like protein | 0.0165 |
| AGDE08063 | -36 | proteasome regulatory non-atp-ase subunit | 0.0174 |
| AGDE00995 | -3 | folate/biopterin transporter | 0.0178 |
| AGDE06960 | -6 | dynein light chain | 0.0192 |
| AGDE00596 | -3 | arginase | 0.0193 |
| AGDE06554 | -3 | nucleolar protein | 0.0198 |
| AGDE07616 | -3 | polyprenol reductase | 0.0203 |
| AGDE00155 | -3 | dihydrolipoamide dehydrogenase | 0.0204 |
| AGDE01259 | -10 | ras-related protein rab-14 | 0.0207 |
| AGDE16806 | -13 | hypothetical protein | 0.0212 |
| AGDE08085 | -11 | protein kinase | 0.0212 |
| AGDE11280 | -13 | conserved hypothetical protein | 0.0221 |
| AGDE09294 | -3 | p450 reductase | 0.0223 |
| AGDE03369 | -35 | vacuolar atp synthase subunit c | 0.0226 |
| AGDE00406 | -4 | chaperonin alpha subunit | 0.0226 |
| AGDE07534 | -4 | phenylalanine-4-hydroxylase | 0.0226 |
| AGDE01335 | -4 | hypothetical protein | 0.0235 |
| AGDE16613 | -5 | conserved hypothetical protein | 0.0242 |
| AGDE06541 | -4 | mitochondrial isocitrate dehydrogenase | 0.0244 |
| AGDE11267 | -3 | squalene monooxygenase-like protein | 0.0246 |
| AGDE07797 | -4 | cytosolic malate dehydrogenase | 0.0252 |
| AGDE01319 | -6 | n-myristoyltransferase | 0.0277 |
| AGDE08574 | -33 | conserved hypothetical protein | 0.0278 |
| AGDE07060 | -95 | serine threonine protein phosphatase type 5 | 0.0283 |
| AGDE02444 | -3 | conserved hypothetical protein | 0.0298 |
| AGDE16763 | -3 | ABC-type transporte system | 0.0313 |
| AGDE07987 | -3 | glycosomal membrane protein | 0.0317 |
| AGDE05390 | -3 | carnitine | 0.0328 |
| AGDE09947 | -11 | haloacid dehalogenase hydrolase | 0.0329 |
| AGDE05545 | -4 | ecotin | 0.0331 |
| AGDE05787 | -23 | u2 splicing auxiliary | 0.0384 |
| AGDE12335 | -21 | conserved hypothetical protein | 0.0384 |
| AGDE05577 | -6 | conserved hypothetical protein | 0.0384 |
| AGDE10917 | -3 | aldehyde dehydrogenase | 0.0384 |
| AGDE06888 | -3 | heat shock protein | 0.0384 |
| AGDE17001 | -4 | conserved hypothetical protein | 0.0392 |
| AGDE10708 | -4 | lathosterol oxidase-like protein | 0.0395 |
| AGDE00061 | -3 | conserved hypothetical protein | 0.0400 |
| AGDE01243 | -6 | rna polymerase b subunit rpb8 | 0.0401 |
| AGDE10382 | -3 | protein kinase | 0.0404 |
| AGDE10777 | -20 | conserved hypothetical protein | 0.0404 |
| AGDE01232 | -3 | 60s ribosomal protein l12 | 0.0420 |
| AGDE11341 | -44 | cytochrome c oxidase assembly protein | 0.0425 |
| AGDE06734 | -26 | iron superoxide dismutase | 0.0457 |
| AGDE09251 | -3 | enolase | 0.0458 |
| AGDE11134 | -3 | heat shock protein atpase subunit | 0.0479 |
| AGDE02848 | -3 | acetyl- synthetase | 0.0482 |
| AGDE11243 | -3 | dihydrolipoamide dehydrogenase | 0.0496 |

AGDE: *Angomonas deanei* sequence feature (SRA Accession: PRJNA279893).

TABLE III

Differential gene expression between *Angomonas deanei* aposymbiotic (APO) and wild type (WT) strains. List of gene transcripts up regulated in APO strain

| Feature ID | Fold change | Gene function (Annotation) | FDR p-value |
|---|---|---|---|
| AGDE02714 | 52 | ribosomal protein l1a | 9.2E-21 |
| AGDE03418 | 340 | hypothetical protein | 1.8E-18 |
| AGDE10054 | 77 | serine peptidase | 2.7E-18 |
| AGDE04136 | 41 | heat shock protein 100 | 3.2E-12 |
| AGDE16735 | 7 | metacaspase | 4.0E-08 |
| AGDE03792 | 53 | mitochondrial tryparedoxin | 1.6E-06 |
| AGDE04435 | 18 | conserved hypothetical protein | 4.9E-06 |
| AGDE11862 | 31 | vacuolar protein sorting-associated protein 4 | 7.7E-06 |
| AGDE16940 | 7 | conserved hypothetical protein | 3.1E-05 |
| AGDE16861 | 5 | hypothetical protein | 4.9E-05 |
| AGDE12000 | 9 | protein kinase | 0.0001 |
| AGDE08977 | 165 | conserved hypothetical protein | 0.0001 |
| AGDE09083 | 7 | branched-chain amino acid aminotransferase | 0.0002 |
| AGDE16676 | 4 | conserved hypothetical protein | 0.0003 |
| AGDE00528 | 18 | conserved hypothetical protein | 0.0004 |
| AGDE05202 | 8 | minichromosome maintenance complex | 0.0004 |
| AGDE07150 | 10 | vacuolar protein sorting-associated protein 4 | 0.0005 |
| AGDE08997 | 16 | conserved hypothetical protein | 0.0005 |
| AGDE05706 | 8 | ribosomal protein l1a | 0.0005 |
| AGDE11571 | 7 | translation factor SUI1 | 0.0005 |
| AGDE14375 | 9 | circumsporozoite protein | 0.0006 |
| AGDE17158 | 4 | hypothetical protein | 0.0008 |
| AGDE16978 | 7 | conserved hypothetical protein | 0.0013 |
| AGDE10823 | 4 | protein kinase | 0.0018 |
| AGDE16514 | 4 | conserved hypothetical protein | 0.0019 |
| AGDE04133 | 9 | udp-glucose pyrophosphorylase | 0.0029 |
| AGDE16371 | 4 | conserved hypothetical protein | 0.0035 |
| AGDE16675 | 5 | conserved hypothetical protein | 0.0047 |
| AGDE11076 | 4 | propionyl-coa carboxylase beta chain | 0.0048 |
| AGDE14602 | 7 | conserved hypothetical protein | 0.0052 |
| AGDE16372 | 4 | conserved hypothetical protein | 0.0054 |
| AGDE10194 | 10 | conserved hypothetical protein | 0.0056 |
| AGDE10373 | 26 | conserved hypothetical protein | 0.0069 |
| AGDE00415 | 4 | conserved hypothetical protein | 0.0086 |
| AGDE16447 | 4 | pre-mrna splicing factor | 0.0088 |
| AGDE07680 | 10 | conserved hypothetical protein | 0.0092 |
| AGDE15874 | 5 | ATP-binding cassette protein subfamily A | 0.0132 |
| AGDE08430 | 5 | branched-chain amino acid aminotransferase | 0.0138 |
| AGDE15539 | 4 | GMP synthase | 0.0138 |
| AGDE07170 | 3 | propionyl-coa carboxylase beta chain | 0.0142 |
| AGDE16950 | 6 | PadR family transcriptional regulator | 0.0145 |
| AGDE00082 | 31 | dihydrolipoamide dehydrogenase | 0.0149 |
| AGDE13187 | 4 | glycosyltransferase group 1 family | 0.0149 |
| AGDE00804 | 7 | hypothetical protein | 0.0159 |
| AGDE16626 | 4 | conserved hypothetical protein TTHERM | 0.0159 |
| AGDE04004 | 5 | conserved hypothetical protein | 0.0166 |
| AGDE08637 | 4 | rna polymerase ii largest subunit | 0.0172 |
| AGDE13234 | 3 | conserved hypothetical protein | 0.0178 |
| AGDE02425 | 78 | conserved hypothetical protein | 0.0196 |
| AGDE15579 | 4 | conserved hypothetical protein | 0.0212 |
| AGDE04400 | 5 | propionyl-coa carboxylase beta chain | 0.0221 |
| AGDE13758 | 4 | nonsense mRNA reducing factor | 0.0232 |
| AGDE14376 | 3 | conserved hypothetical protein TGME | 0.0239 |
| AGDE15677 | 3 | conserved hypothetical protein | 0.0251 |
| AGDE08441 | 4 | endo exonuclease mre11 | 0.0270 |
| AGDE10321 | 4 | amastin | 0.0277 |
| AGDE15693 | 3 | conserved hypothetical protein | 0.0277 |
| AGDE13224 | 4 | conserved hypothetical protein | 0.0301 |
| AGDE09994 | 23 | conserved hypothetical protein | 0.0325 |
| AGDE13223 | 3 | conserved hypothetical protein | 0.0365 |
| AGDE15197 | 68 | conserved hypothetical protein | 0.0370 |
| AGDE03972 | 6 | cytochrome-b5 reductase | 0.0370 |
| AGDE10457 | 3 | hypothetical protein | 0.0375 |
| AGDE17176 | 7 | glutamamyl carboxypeptidase | 0.0384 |
| AGDE12339 | 6 | conserved hypothetical protein | 0.0392 |
| AGDE02016 | 4 | conserved hypothetical protein | 0.0392 |
| AGDE16448 | 4 | formin | 0.0399 |
| AGDE16566 | 3 | neurohypophysial n-terminal domain containing protein | 0.0401 |
| AGDE03338 | 3 | prostaglandin f synthase | 0.0448 |
| AGDE15358 | 3 | hypothetical protein | 0.0456 |
| AGDE12076 | 3 | conserved hypothetical protein | 0.0500 |

AGDE: *Angomonas deanei* sequence feature (SRA Accession: PRJNA279893).

TABLE IV

Enrichment of genes from functional biological process category downregulated in aposymbiotic *Angomonas deanei* strain. Gene ontology (GO)terms of biological process and the number of genes involved in each function are listed according to the p-value

| | Biological processes category GO terms | Description | Number of genes | p-value |
|---|---|---|---|---|
| 1 | 55114 | oxidation-reduction process | 418 | 0.0000 |
| 2 | 15991 | ATP hydrolysis coupled proton transport | 75 | 0.0000 |
| 3 | 6108 | malate metabolic process | 16 | 0.0002 |
| 4 | 6096 | glycolysis | 48 | 0.0014 |
| 5 | 6099 | tricarboxylic acid cycle | 35 | 0.0026 |
| 6 | 6694 | steroid biosynthetic process | 8 | 0.0029 |
| 7 | 46034 | ATP metabolic process | 9 | 0.0051 |
| 8 | 6102 | isocitrate metabolic process | 5 | 0.0066 |
| 9 | 6561 | proline biosynthetic process | 6 | 0.0067 |
| 10 | 44262 | cellular carbohydrate metabolic process | 16 | 0.0090 |
| 11 | 7165 | signal transduction | 11 | 0.0110 |
| 12 | 6633 | fatty acid biosynthetic process | 46 | 0.0139 |
| 13 | 8654 | phospholipid biosynthetic process | 7 | 0.0219 |
| 14 | 6091 | generation of precursor metabolites and energy | 7 | 0.0303 |
| 15 | 6007 | glucose catabolic process | 6 | 0.0330 |
| 16 | 6166 | purine ribonucleoside salvage | 5 | 0.0370 |
| 17 | 6167 | AMP biosynthetic process | 5 | 0.0370 |
| 18 | 6544 | glycine metabolic process | 8 | 0.0397 |

TABLE V

Enrichment of genes from functional biological process category upregulated in aposymbiotic *Angomonas deanei* strain. Gene ontology(GO) terms of biological process and the number of genes involved in each function are listed according to the p-value

| | Biological processes category GO terms | Description | Number of genes | p-value |
|---|---|---|---|---|
| 1 | 6508 | proteolysis | 95 | 0.0000 |
| 2 | 9987 | cellular process | 59 | 0.0002 |
| 3 | 10506 | regulation of autophagy | 5 | 0.0002 |
| 4 | 9081 | branched-chain amino acid metabolic process | 7 | 0.0023 |
| 5 | 7018 | microtubule-based movement | 93 | 0.0032 |
| 6 | 8652 | cellular amino acid biosynthetic process | 18 | 0.0045 |
| 7 | 6270 | DNA replication initiation | 14 | 0.0045 |
| 8 | 44267 | cellular protein metabolic process | 11 | 0.0047 |
| 9 | 44237 | cellular metabolic process | 70 | 0.0054 |
| 10 | 6351 | transcription, DNA-dependent | 24 | 0.0169 |
| 11 | 6260 | DNA replication | 38 | 0.0246 |
| 12 | 6310 | DNA recombination | 10 | 0.0246 |
| 13 | 6264 | mitochondrial DNA replication | 5 | 0.0309 |
| 14 | 6281 | DNA repair | 24 | 0.0336 |
| 15 | 44260 | cellular macromolecule metabolic process | 8 | 0.0340 |
| 16 | 35556 | intracellular signal transduction | 9 | 0.0351 |
| 17 | 6139 | nucleobase-containing compound metabolic process | 14 | 0.0363 |
| 18 | 6400 | tRNA modification | 10 | 0.0382 |
| 19 | 9396 | folic acid-containing compound biosynthetic process | 8 | 0.0453 |
